# Seroprevalence of respiratory viral pathogens of indigenous calves in Western Kenya

**DOI:** 10.1016/j.rvsc.2016.08.010

**Published:** 2016-10

**Authors:** R. Callaby, P. Toye, A. Jennings, S.M. Thumbi, J.A.W. Coetzer, I.C. Conradie Van Wyk, O. Hanotte, M.N. Mbole-Kariuki, B.M.de.C. Bronsvoort, L.E.B. Kruuk, M.E.J. Woolhouse, H. Kiara

**Affiliations:** a Centre for Immunity, Infection and Evolution, University of Edinburgh, Ashworth Laboratories, King's Buildings, West Mains Road, Edinburgh EH9 3JT, UK; b James Hutton Institute, Craigiebuckler, Aberdeen AB15 8QH, UK; c International Livestock Research Institute, P.O. Box 30709, Nairobi 00100, Kenya; d The Farm Animal Practice, University of Edinburgh, Easter Bush, Edinburgh EH25 9RG, UK; e Paul G Allen School for Global Animal Health, Washington State University, Pullman, WA 99164-7079, USA; f Department of Veterinary Tropical Diseases, Faculty of Veterinary Science, University of Pretoria, Private bag X04, Onderstepoort, South Africa; g School of Life Science, University of Nottingham, University Park, Nottingham NG7 2RD, UK; h The Roslin Institute, Easter Bush, University of Edinburgh, Roslin, Midlothian EH25 9RG, UK; i Division of Evolution, Ecology & Genetics, Research School of Biology, The Australian National University, Canberra ACT 0200, Australia; j Institute of Evolutionary Biology, University of Edinburgh, Ashworth Laboratories, Kings Buildings, West Mains Road, Edinburgh EH9 3JT, UK

**Keywords:** Cattle, Bovine respiratory disease complex, Infectious bovine rhinotracheitis, Bovine parainfluenza virus type 3, Bovine viral diarrhoea virus, Zebu

## Abstract

Most studies of infectious diseases in East African cattle have concentrated on gastro-intestinal parasites and vector-borne diseases. As a result, relatively little is known about viral diseases, except for those that are clinically symptomatic or which affect international trade such as foot and mouth disease, bluetongue and epizootic haemorrhagic disease. Here, we investigate the seroprevalence, distribution and relationship between the viruses involved in respiratory disease, infectious bovine rhinotracheitis virus (IBR), bovine parainfluenza virus Type 3 (PIV3) and bovine viral diarrhoea virus (BVDV) in East African Shorthorn Zebu calves. These viruses contribute to the bovine respiratory disease complex (BRD) which is responsible for major economic losses in cattle from intensive farming systems as a result of pneumonia. We found that calves experience similar risks of infection for IBR, PIV3, and BVDV with a seroprevalence of 20.9%, 20.1% and 19.8% respectively. We confirm that positive associations exist between IBR, PIV3 and BVDV; being seropositive for any one of these three viruses means that an individual is more likely to be seropositive for the other two viruses than expected by chance.

## Highlights

• IBR, PIV3 and BVDV are circulating in East Africa.
• Calves experience similar risks of infection for IBR, PIV3, and BVDV with a seroprevalence of 20.9%, 20.1% and 19.8%.

## 1 Introduction

Most studies of infectious diseases in East African cattle have concentrated on gastro-intestinal parasites and vector-borne diseases. As a result, relatively little is known about viral diseases, except for those that are clinically symptomatic or which affect international trade such as foot and mouth disease, bluetongue and epizootic haemorrhagic disease (Bronsvoort et al., 2003, Toye et al., 2013). Yet, in the rest of the world, other viral diseases are known to have a large impact upon the livestock industry. For example, bovine respiratory disease complex (BRD) is responsible for major economic losses in cattle from intensive farming systems as a result of pneumonia (Bowland and Shewen, 2000). Furthermore, studies of intensively farmed feedlot and beef cattle in North America have shown that individuals infected with BRD exhibit a decreased growth rate and some individuals show signs of clinical illness (Gardner et al., 1999, Schneider et al., 2009, White and Renter, 2009).

There are many factors which contribute to the BRD, including stress, management practices and biological agents (Snowder et al., 2006, Taylor et al., 2010). Viruses have been implicated in contributing to BRD by causing lesions in the bovine respiratory tract and/or impairing the clearance of bacteria from the lower respiratory tract (Coetzer and Tustin, 2004). Some of the viruses contributing to BRD include infectious bovine rhinotracheitis virus (IBR), bovine parainfluenza virus type 3 (PIV3) and bovine viral diarrhoea virus (BVDV). Each of these viruses has specific clinical signs, consequences and economic importance (see Appendix). In addition, both IBR and BVDV are immunosuppressive (Hutchings et al., 1990, Koppers-Lalic et al., 2001, Roth et al., 1986, Wellenberg et al., 2002). Numerous epidemiological studies have reported positive associations between these viruses (Fulton et al., 2000, Martin and Bohac, 1986) and the relationship has been reviewed since the 1980s (Yates, 1982). Although these viruses have been documented in many parts of the world (Coetzer and Tustin, 2004), little is known about their distribution within sub-Saharan Africa, particularly in East African extensive farming systems.

The purpose of this study is to improve current knowledge on the seroprevalence and distribution of IBR, PIV3, and BVDV in cattle in sub-Saharan Africa by estimating the seroprevalence to these viruses in East African shorthorn zebu calves from Western Kenya using data gathered by the Infectious Diseases of East Africa Livestock (IDEAL) project. We aim to quantify the associations between these viruses within this study population.

## 2 Material and Methods

### 2.1 Study Population

The study reported here uses data gathered as part of the Infectious Diseases of East Africa Livestock (IDEAL) project, the design of which has previously been reported by Bronsvoort et al. (2013). Briefly, the IDEAL project was an intensive cohort study of 548 indigenous shorthorn zebu calves from 3 to 7 days old which were followed during their first year of life between October 2007 and September 2010.

Calves were selected using a stratified two-stage random cluster study design. In the first stage, 20 sublocations (the smallest administrative unit in Kenya) were selected from 5 agro-ecological zones, in a radius of 45 km from the town of Busia for logistical reasons. This area is dominated by smallholder mixed crop-livestock production systems, with an average farm size of 2 ha and about 5 cattle per farm (Bronsvoort et al., 2013). During the second sampling stage, 28 calves from each sublocation were recruited. To be recruited into the study, calves were aged between 3 and 7 days old, their dam had to have been on the farm for at least one year, the calf was not a result of artificial insemination and the herd should have been under open grazing management. Only one dam and calf from a farm could be in the study at any time. This study focuses on the 455 calves which survived until 51 weeks old.

### 2.2 Data Collection

At the recruitment visit, the calf's owner completed a questionnaire. This questionnaire collected environmental information about the farm, other livestock, water sources and animal husbandry practices. Calf locations were geo-referenced using hand-held GPS devices (Garmin 12, Garmin Kansas, USA). During the study calves were weighed (measured to the nearest 0.5 kg) and the dam's girth was measured (measured in cm) and the calves had biological samples taken for laboratory analysis.

### 2.3 Laboratory Analysis

#### 2.3.1 Viruses

Jugular vein blood samples were collected in plain Vacutainer™ (Becton Dickinson, England) tubes. Blood samples were allowed to clot, the serum was recovered and aliquots were stored at − 20 °C, until serological analysis could take place.

Serological samples taken from calves at 51 weeks of age were screened using SVANOVIR kits obtained from Svanova Biotech AB (Uppsala, Sweden) to identify antibodies specific to the following viruses: a) infectious bovine rhinotracheitis (IBR); b) bovine viral diarrhoea virus (BVDV) antibody and c) bovine parainfluenza virus type 3 (PIV3). In addition, the sera were screened for the presence of BVDV antigens using kits obtained from IDEXX (Montpellier SAS, France). All the kits (apart from the BVDV antigen kit) were designed to detect virus specific IgG antibodies in serum using a procedure based upon a solid phase indirect Enzyme Linked Immunosorbent Assay (Indirect ELISA). All assays were performed and analysed according to the manufacturer's instructions using single wells. There is no evidence for cross-reactivity between the tests.

The SVANOVIR ELISA tests are based on the measurement of corrected optical density (OD_corr_). To calculate the OD_corr_, values in wells coated with a particular antigen are corrected by subtracting the OD values of corresponding wells containing the control antigen (OD_test antigen_ − OD_control_ = OD_corr_). The Percent Positivity (PP) values are calculated as follows:

$$\mathrm{Percent}\;\mathrm{Positivity}=\frac{\mathrm{Test}\;\mathrm{Sample}\left({\mathrm{OD}}_{\text{corr}}\right)\times 100}{\mathrm{Positive}\;\mathrm{Control}\left({\mathrm{OD}}_{\text{corr}}\right)}$$

IDEXX ELISA test results are interpreted using a similar index called the “sample to positive control percentage” (S/P%), which was calculated according to the manufacturer's instructions for the test.

To convert the continuous PP (or S/P%) values into a serostatus which is a binary seropositive/seronegative outcome, the PP (or S/P%) values were interpreted according to the manufacturer's cut-offs specified in the appendix. Justification for grouping the inconclusive IBR results with the IBR seropositive individuals is presented in the appendix. Therefore, seropositive/seronegative means antibodies to the antigen of interest are detectable/not detectable in the blood of the host, whereas seroconversion is the movement from a seronegative to a seropositive state.

### 2.4 Statistical Analysis

#### 2.4.1 Seroprevalence of the Viruses

The crude seroprevalence for each virus was calculated using the *epicalc* package in R v.2.15.2 (Chongsuvivatwong, 2012). The weighted adjusted seroprevalence for each virus was calculated using the R *survey* package (Lumley, 2012). The weighting adjusted for the number of breeding dams in each sublocation. Sublocation-specific seroprevalence was mapped using ArcGIS.

#### 2.4.2 Associations between Viruses

The association between the three viruses was assessed using generalised linear mixed models (GLMM) fitted with a logit link function and binomial errors and a Laplace approximation to the maximum likelihood estimation in R v.2.15.2 using the *lme4* package (Bates et al., 2014). We investigate the association between the serostatus of virus A at 51 weeks old with serostatus of the other viruses at 51 weeks with the following model structure:

$$\begin{array}{l} \log it\left(\mathrm{Virus}\;A\;\mathrm{serostatu}s_{i}\right)\\ \;=\alpha +\beta_{1}\;\mathrm{Virus}\;B\;\mathrm{serostatu}s_{i}+\beta_{2}\;\mathrm{Virus}\;C\;\mathrm{serostatu}s_{i}+\mathrm{Sublocatio}n_{i} \end{array}$$

where *α* is the intercept. *Virus X serostatus*_*i*_ is the serostatus of the calf (*i*) at 51 weeks of age for each virus, and is included in the models as a fixed effect (*β*). Sublocation (*Sublocation*, 20 levels) is included in the model as a random effect (*b*) to account for the study design and environmental similarity between calves clustered into each sublocation.

A separate model was constructed for each virus. Viruses were said to be co-distributed if they occurred in an individual more often than expected by chance. The same patterns of association were examined using continuous measures of PP values instead of serostatus (see the Appendix). Interaction between the viruses was also considered, however including an interaction term between the viruses did not improve the fit of any of the models.

Following construction of the virus-only model, environmental confounders associated with the virus with a *p* < 0.2 in the univariate analysis (listed in the Appendix), which may affect respiratory pathogens or contact between calves, were added into each of the models. Backwards stepwise selection was then used to remove variables to produce the most parsimonious model.

By using a two stage statistical approach it was possible to examine whether the associations between being seropositive for the different viruses was due to coinfection or if the associations were being driven by a joint underlying correlation such as a shared environmental factor or calf characteristic.

## 3 Results

### 3.1 Seroprevalence of the Viruses

**Fig. 1:**
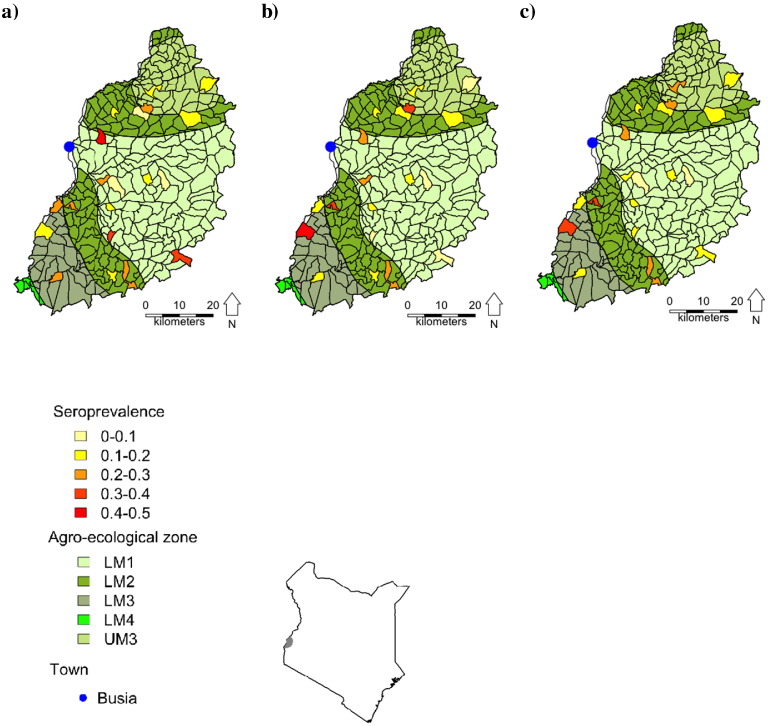
Map of western Kenya showing the distribution of calves seropositive for a) IBR; b) PIV3 and c) BVDV antibody at 51 weeks of age. The map also shows the five agro-ecological zones coloured different shades of green and the 20 sublocations in the study area are shaded according the observed seroprevalence for each virus within them. The blue circle indicates the location of the project laboratory in Busia. The small insert map shows the study area, in grey, in relation to the whole of Kenya.

The seroprevalence at 51 weeks was similar for each virus; IBR had an adjusted seroprevalence of 20.9% whilst PIV3 and BVDV antibodies had adjusted seroprevalences of 20.1% and 19.8%, respectively (Table 1). We observed that 111 (24.4%), 35 (7.7%) and 23 (5.1%) calves were seropositive for 1, 2 or 3 viruses, respectively. Two hundred and eighty five (62.6%) calves were seronegative for all three virus antibodies. BVDV antigen was not detected in the serum of any animal. The seroprevalence of the viruses in each sublocation is plotted in Fig. 1.

### 3.2 Associations between Viruses

**Fig. 2:**
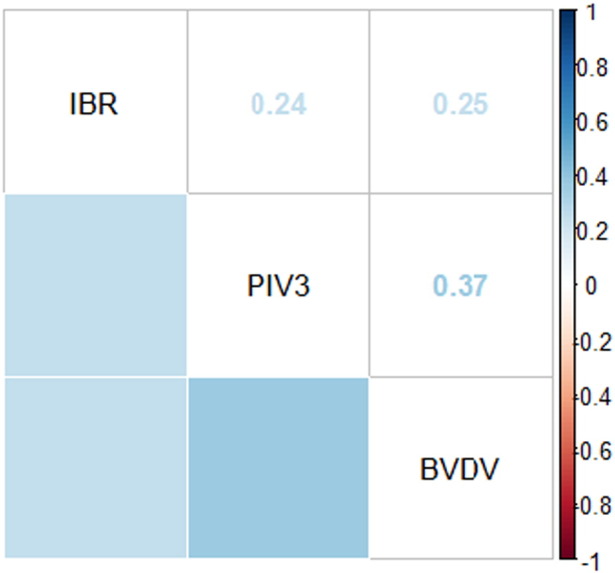
Correlation matrix showing the correlation between seroconversion to IBR, PIV3 and BVDV antibody without correcting for any other variables.

The virus-only GLMMs indicated that IBR, PIV3 and BVDV were co-distributed (Table 2). There was an increased risk of an individual being seropositive for one of these viruses, if the calf was also seropositive for one or both of the other viruses (Table 2). In addition, a positive correlation was observed between the three viruses (Fig. 2). The same pattern of results were observed using PP value as a continuous variable (results not shown).

Inclusion of environmental confounders in the virus-only models did not affect the relationship observed between the serostatus of the three viruses; the most parsimonious model for all three viruses was the one which excluded all environmental variation (Table 2). Even in the univariate analysis, all of 17 environmental risk factors considered had a p value > 0.01 and did not alter the direction or magnitude of the associations with any of the three viruses (see Appendix).

## 4 Discussion

This study aimed to describe the prevalence and association of viruses involved in the bovine respiratory disease complex (BRD) in Western Kenya. We have shown that IBR, PIV3 and BVDV all have an estimated seroprevalence of around 20%. The observed seroprevalence of IBR is within the range (16%–54%) that McDermott et al. (1997) estimated for three districts in Kenya (not including the Busia district) in 1991–1992. However the seroprevalence of the three viruses is lower than that observed in traditionally managed herds in Zambia, which range from 42%–76% (Ghirotti et al., 1991) and lower than the prevalence's observed smallholder farms in coastal Kenya (BVDV prevalence = 45.8%; IBR prevalence = 28.6%; Kenyanjui et al. (2007)). All three viruses are transmitted via secretions or aerosols, in addition, vertical transmission of BVDV can also occur. Therefore one explanation for the difference observed is that the herd sizes in the Zambian study range from 20 to 100 cattle (Ghirotti et al., 1991). IBR, PIV3 and BVDV are observed at higher prevalences in larger herd sizes and they are more common in intensively farmed animals, where there is a high level of contact between individuals (Snowder et al., 2006). Cattle in the IDEAL project are extensively farmed, with a median herd size of 5 and so the risk of contact between a susceptible individual with an infected or persistently infected individual is lower. Furthermore, the cattle in Ghirotti et al. (1991) included individuals aged 3 months to adults, whereas the IDEAL calves were aged 51 weeks old, so there may also be differences in age-related seroprevalence. Moreover, both Ghirotti et al. (1991) and Kenyanjui et al. (2007) used the virus neutralization tests instead of ELISA tests, therefore differences in test sensitivity and specificity may also be contributing to the differences observed in prevalence (Graham et al., 1998). In addition, no BVDV antigen positive calves were identified in this study, suggesting that there are no persistently infected individuals (Brock, 2003).

Cross-reactivity may occur between a virus and its related viruses. For example, BVDV is cross-reactive with other pestiviruses such as Classical Swine Fever and Border Disease Virus of sheep; IBR is cross-reactive with four herpesviruses from other animals including goats and buffalo; and PIV3 cross-reacts with human strains of the virus (Coelingh et al., 1986, Handel et al., 2011, Lyaku et al., 1992). However, since none of the above viruses are expected to be found in cattle in western Kenya, the majority of seropositivity was likely due to exposure to the virus of interest.

In accordance with numerous other epidemiological studies, but in a previously unstudied setting, this analysis has found that IBR, PIV3 and BVDV are associated (Durham and Hassard, 1990, Fulton et al., 2000, Martin and Bohac, 1986). Inclusion of environmental confounders into the models quantifying the relationship between the serostatus of the three viruses had little effect on the association observed between them. Other studies have suggested that at the herd level the main risk factors for BRD are the production type, herd size, housing and management practices such as animal movement and hygiene (Gay and Barnouin, 2009). Risk factors for IBR include increased movement into the herd and distance to neighbouring farms, which increases the risk of infection through contacts with infected individuals (van Schaik et al., 1998). Risk factors for PIV3 and BVDV includes age (Figueroa-Chavez et al., 2012). In addition, the presence of a persistently infected individual can increase BVDV risk (Mainar-Jaime et al., 2001). Since all the calves in this study were the same age when testing took place and sublocation was fitted as a random effect, this variation was removed from the study.

To conclude, this study shows that the viruses IBR, PIV3 and BVDV are co-circulating in East African shorthorn zebu calves in Western Kenya. We identified positive associations occurring between IBR, PIV3 and BVDV in a previously unstudied setting. Further studies are needed to identify the long-term impact of these viruses on cattle productivity and their interactions with other parasites.

## Ethical and Regulatory Guidelines

The IDEAL project received approval by the University of Edinburgh Ethics Committee (reference number OS 03–06), and the Animal Care and Use Committee of the International Livestock Research Institute. All participating farmers gave informed consent in their native language prior to recruiting their animals into the study.

## Figures and Tables

**Table 1:** Crude and adjusted seroprevalence of each virus. Seroprevalence is followed by the 95% confidence interval in brackets. IBR = infectious bovine rhinotracheitis; PIV3 = bovine parainfluenza virus type 3; BVDV = bovine viral diarrhoea virus.

Virus	Number of seropositive calves	Number of calves tested	Crude Seroprevalence (95% CI)	Adjusted Seroprevalence (95% CI)
IBR	91^a^	455	20.00 (16.42–23.98)	20.90 (16.41–25.39)
PIV3	80	455	17.62 (14.23–21.44)	20.08 (15.52–24.63)
BVDV (antibody test)	79	454	17.36 (13.99–21.16)	19.76 (15.17–24.36)

a 66 of the IBR inconclusive calves (according to the manufacturers cut-offs) were classified as seropositive following the case-case analysis in the appendix.

**Table 2:** Odds ratio and 95% confidence interval from the virus-only analyses of the association between seroconversion to IBR, PIV3 and BVDV.

Response virus	Explanatory virus									Random effect: Sublocation	
	IBR			PIV3			BVDV				
	OR	95% CI	P value	OR	95% CI	P value	OR	95% CI	P value	Variance	SD
IBR	–	–	–	2.77	1.53–5.03	0.001	2.77	1.54–5.00	0.001	0.15	0.38
PIV3	2.76	1.53–4.98	0.001	–	–	–	5.87	3.30–10.46	< 0.001	0.14	0.38
BVDV (antibody test)	2.77	1.56–4.92	0.001	5.98	3.40–10.52	< 0.001	–	–	–	0.00	0.00

## Appendix A Supplementary Materials

Supplementary materials. Image 1
