# Supplementary material for: Seroprevalence of respiratory viral pathogens of indigenous calves in Western Kenya
Source: Res Vet Sci. 2016 Oct;108:120–4. doi: 10.1016/j.rvsc.2016.08.010 (PMC5040193; doi:10.1016/j.rvsc.2016.08.010)
Supplement: Supplementary file 1 — Supplementary materials. [file mmc1.docx]

Table A.1: Description of the aetiology, epidemiology and pathogenesis of IBR, PIV3 and BVDV

|  | **Infectious Bovine Rhinotracheitis (IBR)** | **Bovine Parainfluenza Virus Type 3 (PIV3)** |
| --- | --- | --- |
| Aetiology | Genus: *Varicellavirus*  Family: Herpeviridae | Genus: *Paramyxovirus*  Family: Paramyxoviridae |
| Epidemiology | Spread by aerosols and secretions. Latent infection can occur. | Spread by aerosols. No persistently infected individuals reported. |
| Pathogenesis | Local lesions in mucus membranes. Virus multiples in the  epithelial cells of the upper respiratory tract and then spreads to the lower respiratory tract in uncomplicated infections. The replicating virus causes destruction of the epithelial cells and alteration of the mucociliary clearance mechanisms. | Primarily infected cells are the epithelial cells of the trachea, bronchi and alveoli. Necrosis of ciliated epithelium interferes with the mucociliary clearance of the airways. No evidence being immunosuppressive. Predisposes lung tissue to bacterial invasion. Replicates in alveolar macrophages. |
| Clinical signs | Fever, increased respiratory rate, inappetence, coughing, nasal discharge, drop in milk production, hyperaemia and small foci of necrosis of the nasal passage, excessive salivation. Course of disease is variable among individuals and secondary bacterial infection will increase the severity and duration of clinical signs. | Mild or subclinical. Development of disease depends upon interaction with other infectious and environmental factors. |
| References | [Coetzer and Tustin (2004)](#_ENREF_3) | [Coetzer and Tustin (2004)](#_ENREF_3) |

Continued on next page

**Table A.1: Continued from previous page**

|  | **Bovine Viral Diarrhoea Virus (BVDV)** |
| --- | --- |
| Aetiology | Genus: *Pestivirus*  Family: Flavividae |
| Epidemiology | Spread by secretions, horizontal and vertical transmission can occur. Immuno-tolerant persistently infected individuals reported |
| Pathogenesis | Replicates in the nasal mucosa and the tonsils. Spreads to regional lymph nodes and the rest of the body. Induces immunosuppression. |
| Clinical signs | Depends on the host, environment, epidemiological and viral factors. May result in one of three disease syndromes: Bovine viral diarrhoea, mucosal disease or embryonal/foetal disease. Some of the clinical signs include abortions, fever, inappentance, diarrhoea, and ulceration of the gastrointestinal tract. |
| References | [Coetzer and Tustin (2004)](#_ENREF_3) |

Table A.2: Manufacturers cut-offs for the interpretation of ELISA percent positivity (PP) values for antibodies to IBR, PIV3 and BVDV and manufacturer cut-off for the interpretation of sample to positive control percentage (S/P%) values for BVDV antigen

| **Test** | **Manufacturer** | **Manufacturers Cut-offs** | |
| --- | --- | --- | --- |
|  |  | **PP** | **Interpretation** |
| Infectious bovine rhinotracheitis antibody | SVANOVIR | < 8 | Negative |
|  |  | 8-11 | Doubtful |
|  |  | > 12 | Positive |
| Bovine parainfluenza virus type 3 | SVANOVIR | < 10 | Negative |
|  |  | > 10 | Positive |
| Bovine virus diarrhoea virus antibody | SVANOVIR | < 14 | Negative |
|  |  | > 14 | Positive |
| Bovine virus diarrhoea virus antigen | IDEXX | < 0.300* | Negative |
|  |  | > 0.300* | Positive |

*Sample to positive control percentage (S/P%) given rather than PP

## Case-case analysis of IBR doubtful and seropositive calves

Since the manufacturer’s cut-off for IBR classifies individuals into ‘seronegative’, ‘seropositive’ or ‘doubtful’ (Table A.1), it is important to check that there are no differences in outcomes of interest between the seropositive and doubtful individuals if we are to combine them into a binary seropositive/seronegative serostatus variable. A case-case analysis was used to achieve this. The case-case analysis used chi-squared tests to investigate differences between IBR doubtful and seropositive calves in the number which experienced clinical episodes, or were seropositive for BVDV or PIV3. t-tests were used to test for a difference in the mean live body weight at recruitment and at one year old, mean daily weight gain of IBR seropositive and doubtful individuals. This whole process was then repeated to compare IBR doubtful and seronegative calves.

Comparison of IBR seropositive and doubtful individuals revealed that there was no statistically significant difference between the two categories. Both have a similar number of clinical episodes, BVDV and PIV3 seropositive, as well as having a similar mean daily weight gain (Table A.3 and Table A.4). In contrast, comparison of the IBR seronegative and doubtful individuals revealed that there was a significant difference between the two categories. IBR doubtful individuals were more likely to be BVDV and PIV3 seropositive than IBR seronegative individuals, in addition IBR seronegative calves were lighter than IBR doubtful individuals (Table A.5 and Table A.6). These findings imply that the IBR doubtful calves are more similar to the IBR seropositive calves than to the IBR seronegative ones; therefore it is reasonable to combine the IBR seropositive and doubtful categories into one group.

Table A.3: Comparison of categorical traits for IBR *doubtful* (N=66) and IBR *seropositive* (N=25) calves using chi-squared tests.

| **Categorical trait of interest** | **IBR doubtful** | | **IBR seropositive** | | **IBR doubtful vs. IBR seropositive** | | |
| --- | --- | --- | --- | --- | --- | --- | --- |
|  | **N** | **%** | **N** | **%** | **χ^2^** | **d.f.** | **P value** |
| BVDV antibody seronegative | 42 | 46.15 | 16 | 17.58 | 0.001 | 1 | 0.974 |
| BVDV antibody seropositive | 24 | 26.37 | 9 | 9.89 |  |  |  |
| PIV3 seronegative | 42 | 46.15 | 16 | 17.58 | 0.001 | 1 | 0.974 |
| PIV3 seropositive | 24 | 26.37 | 9 | 9.89 |  |  |  |
| Did not experience a clinical episode | 48 | 52.75 | 15 | 16.48 | 1.38 | 1 | 0.240 |
| Experienced a clinical episode | 18 | 19.78 | 10 | 10.99 |  |  |  |
| Did not experience a respiratory clinical episode | 63 | 69.23 | 22 | 24.18 | 1.64 | 1 | 0.200 |
| Experienced a respiratory clinical episode | 3 | 3.30 | 3 | 3.30 |  |  |  |

Table A.4: Comparison of continuous traits for IBR *doubtful* (N=66) and IBR *seropositive* (N=25) calves using t-tests.

| **Continuous Trait of Interest** | **IBR doubtful** | | **IBR seropositive** | | **T** | **d.f.** | **P value** |
| --- | --- | --- | --- | --- | --- | --- | --- |
|  | Mean | SE | Mean | SE |  |  |  |
| Live body weight at recruitment | 20.78 | 0.45 | 18.80 | 0.92 | 1.94 | 36 | 0.060 |
| Live body weight at one year | 69.89 | 2.44 | 63.10 | 3.79 | 1.51 | 45 | 0.138 |
| Average daily weight gain | 0.14 | 0.01 | 0.13 | 0.01 | 0.96 | 46 | 0.342 |

Table A.5: Comparison of categorical traits for IBR *doubtful* (N= 66) and IBR *seronegative* (N=364) calves using chi-squared tests

| **Categorical Trait of Interest** | **IBR doubtful** | | **IBR seronegative** | | **IBR doubtful vs. IBR seronegative** | | |
| --- | --- | --- | --- | --- | --- | --- | --- |
|  | **N** | **%** | **N** | **%** | **χ^2^** | **d.f.** | **P value** |
| BVDV antibody seronegative | 42 | 9.77 | 318 | 73.95 | 23.08 | 1 | <0.001 |
| BVDV antibody seropositive | 24 | 5.58 | 46 | 10.70 |  |  |  |
| PIV3 seronegative | 42 | 9.79 | 316 | 73.66 | 22.17 | 1 | <0.001 |
| PIV3 seropositive | 24 | 5.59 | 47 | 10.96 |  |  |  |
| Did not experience a clinical episode | 48 | 11.16 | 186 | 43.26 | 10.54 | 1 | 0.001 |
| Experienced a clinical episode | 18 | 4.19 | 178 | 41.40 |  |  |  |
| Did not experience a respiratory clinical episode | 63 | 14.65 | 328 | 76.28 | 1.94 | 1 | 0.164 |
| Experienced a respiratory clinical episode | 3 | 0.70 | 36 | 8.37 |  |  |  |

Table A.6: Comparison of continuous traits for IBR *doubtful* (N=66) and IBR (N=364) *seronegative* calves using t-tests.

| **Continuous Trait of Interest** | **IBR doubtful** | | **IBR seronegative** | | **T** | **d.f.** | **P value** |
| --- | --- | --- | --- | --- | --- | --- | --- |
|  | **Mean** | **SE** | **Mean** | **SE** |  |  |  |
| Live body weight at recruitment | 20.78 | 0.45 | 18.95 | 0.19 | 3.77 | 89 | <0.001 |
| Live body weight at one year | 69.89 | 2.44 | 64.45 | 0.90 | 2.09 | 83 | 0.039 |
| Average daily weight gain | 0.14 | 0.01 | 0.13 | 0.002 | 1.40 | 83 | 0.165 |

## Associations between viruses analysed as a continuous variable

Initially the association between viruses was assessed using the percentage positivity (PP) values as a continuous measure in a generalised linear mixed model (GLMM) fitted with a Laplace approximation to the maximum likelihood estimation in R v.2.15.2 using the *lme4* package ([Bates et al., 2014](#_ENREF_1)). PP values were log_10_ transformed and outlying PPs were removed to normalize the data (Figure A.1). We investigate the association between PP values of multiple viruses at 51 weeks old with the PP value of virus A at 51 weeks using the following equation:

$${PP}_{{Virus A}_{i}} =\alpha+\beta_{1}{PP}_{{Virus B}_{i}}+ \beta_{2}{PP}_{{Virus C}_{i}}+ b_{1_{i}}{Sublocation}_{i}$$

where *α* is the intercept. Fixed effects are symbolized by *β*. *PP_Virus X_* is the PP value of the calf (*i*) at 51 weeks of age. Sublocation (*Sublocation*, 20 levels) is included in the model as a random effect (*b*) to account for the study design and environmental similarity between calves clustered into each sublocation. A separate multivariable model was constructed for each virus and backwards-stepwise selection was used to remove viruses. The most parsimonious model was chosen as the final model.

The statistical analysis of the continuous PP values indicated that IBR, PIV3 and BVDV are co-distributed (Table A.7).

| 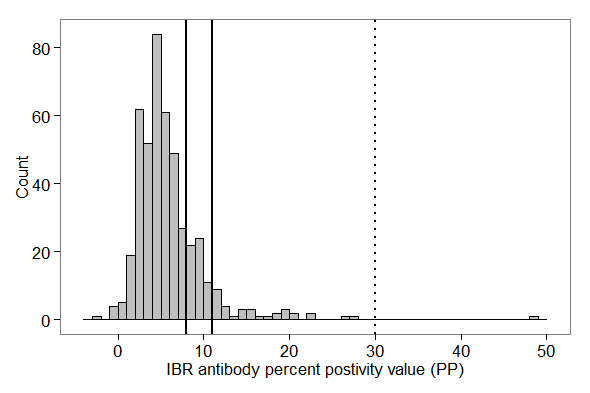 | 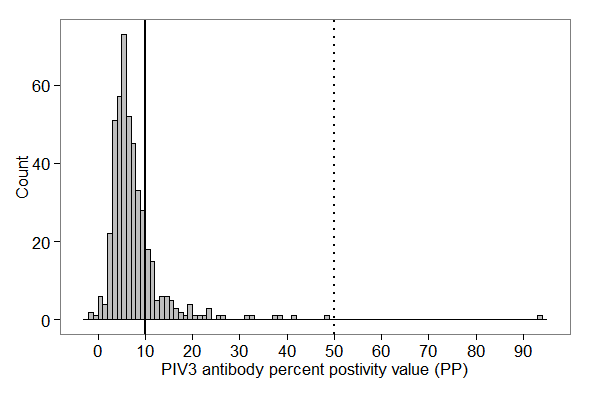 | 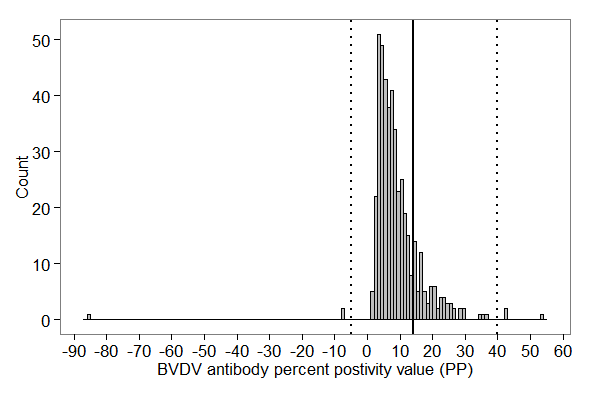 |
| --- | --- | --- |

Figure A.1: Histogram showing the number of individuals against their percentage positivity values (PP) for the IBR, PIV3 and BVDV antibody ELISA tests. The solid vertical line represents the manufacturer’s cut-off, below the dashed line indicates seronegative individuals and above the dashed line represents seropositive individuals. Individuals who fall between the two solid lines in the IBR results are inconclusive. The dotted line represents outliers which were removed in the continuous analysis.

Table A.7: Parameter estimates for the three multivariable analyses of the association between the PP values of IBR, PIV3 and BVDV antibody.

| **Explanatory Virus** | **Response Virus** | **Explanatory Virus** | | | | | |
| --- | --- | --- | --- | --- | --- | --- | --- |
|  |  | **Estimate** | **SE** | **T value** | **OR** | **95% CI** | **P value** |
| **IBR** | **PIV3** | 0.15 | 0.04 | 3.65 | 1.16 | 1.07-1.26 | <0.001 |
|  | **BVDV** | 0.21 | 0.04 | 4.81 | 1.23 | 1.13-1.34 | <0.001 |
| **PIV3** | **IBR** | 0.20 | 0.05 | 3.66 | 1.22 | 1.10-1.36 | <0.001 |
|  | **BVDV** | 0.45 | 0.05 | 9.49 | 1.55 | 1.42-1.70 | <0.001 |
| **BVDV** | **IBR** | 0.24 | 0.05 | 4.81 | 1.28 | 1.15-1.41 | <0.001 |
|  | **PIV3** | 0.39 | 0.04 | 9.46 | 1.48 | 1.36-1.60 | <0.001 |

Table A.8: A list of all the environmental variables which were considered to be biologically plausible confounders examined to investigate if the pathogen-pathogen associations observed were robust to environmental variation

| Farm factors | Farmer’s age, gender, education level, main occupation, land size, number of cattle owned, distance to water, water provision, whether or not the calf was housed with adult cattle |
| --- | --- |
| ‘Farmer quality’ index | As many of the environmental criteria are correlated, we decided to create a farmer quality index. The farmer quality index is a continuous scale from 0 to 6 based upon the number of requirements on the following criteria a farmer met. 0 means that the farmer met none of the criteria, whereas 6 means the farmer met all 6 requirements. The criteria used to form the farmer quality index where: a) The farmer has technical training; b) housing is provided in the form of a Kraal or Stall-Shed or Yard; c) the farmer used supplementary feeding mainly in the form of napier grass, as stated during the recruitment questionnaire; d) the farmer has access to veterinary services; e) the farmer has knowledge of diseases prevalent on the farm; and f) the farmer uses veterinary interventions at some point during the study period in other members of the herd but NOT in the calf (due to the study requirements, Bronsvoort *et al.* ([2013](#_ENREF_2))). Veterinary interventions included the use of antibiotics, antiprotozoals, anthelminthics, insecticides/acaracides, traditional medicine, trypanocidials or vaccines. |
| Environmental factors | Farm altitude (elevation, as categorical variable: <1198m, 1199-1238m, 1239-1269m, >1269m), agro-ecological zone |
| Dam factors | Dam girth (cm), body condition score (a method of assessing the body condition of the dam using a standard 10 point score). |
| Calf factors | Calf sex, heterozygosity and level of European Taurine introgression (the level of introgression consistent with crossing with European breeds less than 5 generations ago, see Mbole-Kariuki *et al.* ([2014](#_ENREF_4)) and Murray *et al.* ([2013](#_ENREF_5)) for more details) |

Table A.9: Association between viruses after accounting for environmental, dam and calf variation. Each line shows a separate model, account for one environmental factor at a time. Inclusion of biologically plausible environmental confounders in the virus-only models did not affect the relationship observed between seroconversion to any of the three viruses.

| **Explanatory Variable** | | | **Response Virus** | | | | | | | | |
| --- | --- | --- | --- | --- | --- | --- | --- | --- | --- | --- | --- |
|  |  |  | **IBR** | | | **PIV3** | | | **BVDV** | | |
|  |  |  | **OR** | **95% CI** | **P value** | **OR** | **95% CI** | **P value** | **BVDV** | **95% CI** | **P value** |
| **Farm Factors** | | |  |  |  |  |  |  |  |  |  |
|  | IBR seropositive | | - | - | - | 2.72 | 1.50-4.91 | 0.001 | 2.76 | 1.55-4.91 | 0.001 |
|  | PIV3 seropositive | | 2.74 | 1.51-4.98 | 0.001 | - | - | - | 5.94 | 3.38-10.45 | <0.001 |
|  | BVDV seropositive | | 2.78 | 1.54-5.03 | 0.001 | 5.83 | 3.27-10.39 | <0.001 | - | - | - |
|  | Farmer age (effect of being greater than 60 years old) | | 1.50 | 0.84-2.66 | 0.170 | 1.10 | 0.58-2.08 | 0.774 | 0.89 | 0.47-1.69 | 0.731 |
|  | IBR seropositive | | - | - | - | 2.77 | 1.53-5.01 | 0.001 | 2.77 | 1.56-4.93 | 0.001 |
|  | PIV3 seropositive | | 2.77 | 1.53-5.02 | 0.001 | - | - | - | 5.98 | 3.40-10.51 | <0.001 |
|  | BVDV seropositive | | 2.79 | 1.55-5.02 | 0.001 | 5.87 | 3.30-10.46 | <0.001 | - | - | - |
|  | Farmer sex (effect of being male) | | 0.65 | 0.39-1.09 | 0.101 | 1.04 | 0.58-1.86 | 0.896 | 1.02 | 0.58-1.79 | 0.955 |
|  | IBR seropositive | | - | - | - | 2.74 | 1.52-4.95 | 0.001 | 2.75 | 1.55-4.88 | 0.001 |
|  | PIV3 seropositive | | 2.77 | 1.52-5.02 | 0.001 | - | - | - | 5.93 | 3.37-10.43 | <0.001 |
|  | BVDV seropositive | | 2.76 | 1.53-4.98 | 0.001 | 5.82 | 3.26-10.36 | <0.001 | - | - | - |
|  | Farmer education level (effect of not having at least primary school level education) | | 1.10 | 0.54-2.21 | 0.794 | 0.84 | 0.38-1.86 | 0.663 | 0.91 | 0.42-1.97 | 0.816 |
|  | IBR seropositive | | - | - | - | 2.81 | 1.55-5.10 | 0.001 | 2.70 | 1.51-4.81 | 0.001 |
|  | PIV3 seropositive | | 2.81 | 1.55-5.12 | 0.001 | - | - | - | 6.00 | 3.40-10.58 | <0.001 |
|  | BVDV seropositive | | 2.70 | 1.50-4.88 | 0.001 | 5.90 | 3.31-10.53 | <0.001 | - | - | - |
|  | Main occupation (effect of not being a farmer) | | 1.66 | 0.85-3.22 | 0.137 | 0.69 | 0.30-1.56 | 0.372 | 1.28 | 0.61-2.71 | 0.513 |
|  | IBR seropositive | | - | - | - | 2.68 | 1.47-4.88 | 0.001 | 2.98 | 1.65-5.38 | 0.000 |
|  | PIV3 seropositive | | 2.71 | 1.48-4.97 | 0.001 | - | - | - | 6.24 | 3.47-11.23 | <0.001 |
|  | BVDV seropositive | | 2.98 | 1.62-5.48 | <0.001 | 6.18 | 3.39-11.27 | <0.001 | - | - | - |
|  | Total are of land used (acres) | | 0.74 | 0.28-1.95 | 0.545 | 0.54 | 0.18-1.61 | 0.271 | 1.74 | 0.62-4.92 | 0.293 |
|  | IBR seropositive | | - | - | - | 2.79 | 1.55-5.02 | 0.001 | 2.65 | 1.48-4.75 | 0.001 |
|  | PIV3 seropositive | | 2.80 | 1.54-5.07 | 0.001 | - | - | - | 6.15 | 3.48-10.87 | <0.001 |
|  | BVDV seropositive | | 2.67 | 1.47-4.86 | 0.001 | 6.07 | 3.39-10.84 | <0.001 | - | - | - |
|  | Total number of cattle on farm (greater than 5) | | 0.81 | 0.48-1.36 | 0.416 | 0.89 | 0.51-1.57 | 0.695 | 1.47 | 0.85-2.53 | 0.164 |
|  | IBR seropositive | | - | - | - | 2.78 | 1.54-5.01 | 0.001 | 2.72 | 1.52-4.86 | 0.001 |
|  | PIV3 seropositive | | 2.79 | 1.53-5.07 | 0.001 | - | - | - | 6.51 | 3.64-11.63 | <0.001 |
|  | BVDV seropositive | | 2.75 | 1.51-4.98 | 0.001 | 6.47 | 3.59-11.67 | <0.001 | - | - | - |
|  | Distance to water <1km | | 0.93 | 0.47-1.85 | 0.843 | 1.72 | 0.74-3.98 | 0.205 | 0.65 | 0.32-1.28 | 0.211 |
|  | Distance to water 1-10Km | | 0.93 | 0.42-2.04 | 0.847 | 2.49 | 0.99-6.26 | 0.053 | 0.36 | 0.15-0.84 | 0.019 |
|  | IBR seropositive | | - | - | - | 2.81 | 1.56-5.07 | 0.001 | 2.67 | 1.49-4.78 | 0.001 |
|  | PIV3 seropositive | | 2.82 | 1.56-5.09 | 0.001 | - | - | - | 6.11 | 3.46-10.8 | <0.001 |
|  | BVDV seropositive | | 2.67 | 1.47-4.83 | 0.001 | 6.02 | 3.37-10.75 | <0.001 | - | - | - |
|  | Water provisioning (animals go to water) | | 1.12 | 0.68-1.85 | 0.656 | 1.18 | 0.69-2.04 | 0.548 | 0.69 | 0.40-1.19 | 0.184 |
|  | IBR seropositive | | - | - | - | 2.26 | 1.05-4.85 | 0.036 | 2.69 | 1.29-5.63 | 0.008 |
|  | PIV3 seropositive | | 2.33 | 1.05-5.19 | 0.039 | - | - | - | 6.49 | 3.18-13.26 | <0.001 |
|  | BVDV seropositive | | 2.69 | 1.24-5.84 | 0.012 | 6.50 | 3.18-13.28 | <0.001 | - | - | - |
|  | Calves housed with adults | | 0.72 | 0.29-1.78 | 0.473 | 1.51 | 0.65-3.53 | 0.337 | 1.29 | 0.56-2.97 | 0.548 |
| **Good or bad farmer indicator scale** | | |  |  |  |  |  |  |  |  |  |
|  | | IBR seropositive | - | - | - | 2.58 | 1.4-4.74 | 0.002 | 2.85 | 1.58-5.12 | <0.001 |
|  | | PIV3 seropositive | 2.58 | 1.4-4.76 | 0.002 | - | - | - | 5.72 | 3.20-10.23 | <0.001 |
|  | | BVDV seropositive | 2.82 | 1.55-5.13 | 0.001 | 5.56 | 3.06-10.09 | <0.001 | - | - | - |
|  | | Good/bad farmer indicator scale | 1.10 | 0.88-1.38 | 0.403 | 0.93 | 0.73-1.18 | 0.566 | 1.20 | 0.94-1.54 | 0.151 |
| **Environmental factors** | | |  |  |  |  |  |  |  |  |  |
|  | | IBR seropositive | - | - | - | 2.77 | 1.53-5.02 | 0.001 | 2.89 | 1.61-5.18 | <0.001 |
|  | | PIV3 seropositive | 2.84 | 1.56-5.18 | 0.001 | - | - | - | 5.97 | 3.34-10.67 | <0.001 |
|  | | BVDV seropositive | 2.89 | 1.59-5.24 | 0.001 | 5.91 | 3.29-10.62 | <0.001 | - | - | - |
|  | | Elevation - 1199-1238m | 1.14 | 0.53-2.44 | 0.741 | 0.80 | 0.38-1.69 | 0.562 | 0.50 | 0.24-1.05 | 0.066 |
|  | | Elevation - 1239-1269m | 1.47 | 0.68-3.18 | 0.326 | 0.41 | 0.18-0.93 | 0.033 | 0.77 | 0.38-1.57 | 0.480 |
|  | | Elevation - >1269m | 1.66 | 0.76-3.64 | 0.203 | 0.83 | 0.37-1.85 | 0.645 | 0.42 | 0.19-0.91 | 0.028 |
|  | | IBR seropositive | - | - | - | 2.54 | 1.40-4.63 | 0.002 | 2.92 | 1.62-5.27 | <0.001 |
|  | | PIV3 seropositive | 2.52 | 1.39-4.55 | 0.002 | - | - | - | 5.61 | 3.15-10.00 | <0.001 |
|  | | BVDV seropositive | 2.89 | 1.59-5.23 | 0.000 | 5.62 | 3.14-10.04 | <0.001 | - | - | - |
|  | | AEZ - LM2 middle | 0.75 | 0.25-2.25 | 0.608 | 0.68 | 0.21-2.16 | 0.514 | 1.75 | 0.6-5.10 | 0.304 |
|  | | AEZ - LM1 | 2.40 | 1.04-5.51 | 0.039 | 1.32 | 0.54-3.25 | 0.543 | 1.15 | 0.45-2.94 | 0.777 |
|  | | AEZ - LM2 south | 1.69 | 0.64-4.48 | 0.291 | 1.08 | 0.37-3.14 | 0.890 | 1.79 | 0.62-5.14 | 0.279 |
|  | | AEZ - LM3 | 1.44 | 0.53-3.89 | 0.470 | 2.46 | 0.90-6.74 | 0.080 | 2.65 | 0.95-7.36 | 0.061 |
| **Dam factors** | | |  |  |  |  |  |  |  |  |  |
|  | | IBR seropositive | - | - | - | 2.63 | 1.44-4.82 | 0.002 | 2.83 | 1.58-5.05 | <0.001 |
|  | | PIV3 seropositive | 2.64 | 1.44-4.84 | 0.002 | - | - | - | 5.88 | 3.32-10.40 | <0.001 |
|  | | BVDV seropositive | 2.84 | 1.57-5.15 | 0.001 | 5.81 | 3.25-10.38 | <0.001 | - | - | - |
|  | | Dam girth at recruitment | 420.03 | 0.01-1.34x10^7^ | 0.253 | 0.00 | 0.00-57.95 | 0.210 | 0.01 | 0-295.40 | 0.349 |
|  | | IBR seropositive | - | - | - | 2.69 | 1.49-4.87 | 0.001 | 2.90 | 1.62-5.18 | <0.001 |
|  | | PIV3 seropositive | 2.69 | 1.48-4.89 | 0.001 | - | - | - | 6.04 | 3.42-10.67 | <0.001 |
|  | | BVDV seropositive | 2.88 | 1.59-5.22 | 0.000 | 5.97 | 3.33-10.68 | <0.001 | - | - | - |
|  | | Dam condition score at recruitment | 1.26 | 0.98-1.63 | 0.074 | 1.13 | 0.85-1.50 | 0.398 | 0.81 | 0.62-1.07 | 0.136 |
| **Calf factors** | | |  |  |  |  |  |  |  |  |  |
|  | | IBR seropositive | - | - | - | 2.75 | 1.53-4.97 | 0.001 | 2.75 | 1.55-4.89 | 0.001 |
|  | | PIV3 seropositive | 2.78 | 1.53-5.05 | 0.001 | - | - | - | 6.01 | 3.42-10.59 | <0.001 |
|  | | BVDV seropositive | 2.76 | 1.53-4.98 | 0.001 | 5.90 | 3.31-10.52 | <0.001 | - | - | - |
|  | | Calf sex (effect of being male) | 1.09 | 0.67-1.78 | 0.731 | 0.90 | 0.52-1.53 | 0.688 | 1.18 | 0.69-2.01 | 0.549 |
|  | | IBR seropositive | - | - | - | 2.80 | 1.55-5.07 | 0.001 | 2.90 | 1.63-5.17 | <0.001 |
|  | | PIV3 seropositive | 2.80 | 1.54-5.09 | 0.001 | - | - | - | 5.92 | 3.34-10.48 | <0.001 |
|  | | BVDV seropositive | 2.88 | 1.59-5.22 | 0.000 | 5.79 | 3.22-10.41 | <0.001 | - | - | - |
|  | | European taurine introgression (moderate) | 0.64 | 0.31-1.34 | 0.241 | 1.00 | 0.46-2.16 | 0.995 | 1.12 | 0.54-2.32 | 0.761 |
|  | | European taurine introgression (substantial) | 0.73 | 0.22-2.37 | 0.599 | 0.55 | 0.14-2.17 | 0.398 | 2.36 | 0.84-6.62 | 0.104 |
|  | | IBR seropositive | - | - | - | 2.79 | 1.54-5.03 | 0.001 | 2.86 | 1.61-5.09 | <0.001 |
|  | | PIV3 seropositive | 2.79 | 1.54-5.06 | 0.001 | - | - | - | 5.78 | 3.27-10.21 | <0.001 |
|  | | BVDV seropositive | 2.82 | 1.56-5.08 | 0.001 | 5.61 | 3.13-10.05 | <0.001 | - | - | - |
|  | | Heterozygosity | 0.22 | 0-1.85x10^5^ | 0.826 | 0.10 | 0-6.25x10^5^ | 0.774 | 3004.86 | 0.00-1.08x10^10^ | 0.298 |

## References

Bates, D., Maechler, M., Bolker, B., Walker, S., 2014. lme4: Linear mixed-effects models using Eigen and S4.

Bronsvoort, B.M.d.C., Thumbi, S., Poole, E., Kiara, H., Auguet, O., Handel, I., Jennings, A., Conradie, I., Mbole-Kariuki, M., Toye, P., Hanotte, O., Coetzer, J., Woolhouse, M.E.J., 2013. Design and descriptive epidemiology of the Infectious Diseases of East African Livestock (IDEAL) project, a longitudinal calf cohort study in western Kenya. BMC Veterinary Research 9, 171.

Coetzer, J.A.W., Tustin, R.C., 2004. Infectious diseases of livestock, 2^nd^ ed. Oxford University Press Southern Africa, Cape Town.

Mbole-Kariuki, M.N., Sonstegard, T., Orth, A., Thumbi, S.M., Bronsvoort, B.M.d.C., Kiara, H., Toye, P., Conradie, I., Jennings, A., Coetzer, K., Woolhouse, M.E.J., Hanotte, O., Tapio, M., 2014. Genome-wide analysis reveals the ancient and recent admixture history of East African Shorthorn Zebu from Western Kenya. Heredity.

Murray, G.G., Woolhouse, M.E.J., Tapio, M., Mbole-Kariuki, M.N., Sonstegard, T.S., Thumbi, S.M., Jennings, A.E., Conradie van Wyk, I., Chase-Topping, M., Kiara, H., Toye, P., Coetzer, K., Bronsvoort, B.M.d.C., Hanotte, O., 2013. Genetic susceptibility to infectious disease in East African Shorthorn Zebu: a genome-wide analysis of the effect of heterozygosity and exotic introgression. BMC Evolutionary Biology 13, 246.
